# Breaking boundaries: A rare case of glioblastoma with uncommon extraneural metastases: A case report and literature review

**DOI:** 10.1016/j.bas.2024.103927

**Published:** 2024-10-10

**Authors:** Erlend Moen Taule, Jorunn Brekke, Hrvoje Miletic, Hege Sætran, Snezana Maric, Ineke HogenEsch, Rupavathana Mahesparan

**Affiliations:** a Department of Biomedicine, University of Bergen, Jonas Lies Vei 91, 5009, Bergen, Norway; b Department of Oncology and Medical Physics, Haukeland University Hospital, Bergen, Norway; c Department of Pathology, Haukeland University Hospital, Bergen, Norway; d Department of Radiology, Haukeland University Hospital, Bergen, Norway; e Department of Neurology, Fonna Hospital Trust, Haugesund, Norway; f Department of Clinical Medicine, University of Bergen Faculty of Medicine and Dentistry, Bergen, Norway; g Department of Neurosurgery, Haukeland University Hospital, Bergen, Norway

**Keywords:** Case report, Glioblastoma, Extraneural metastasis, Neuro-oncology, Molecular biology

## Abstract

**Introduction:**

Extraneural metastases (ENM) from glioblastoma (GBM) remain extremely rare with only a scarce number of cases described in the literature. The lack of cases leads to no consensus on the optimal treatment and follow-up of these patients.

**Research question:**

Do patient or tumor characteristics describe risk factors for ENM in GBM patients, and is it possible to identify mechanisms of action?

**Material and methods:**

This study presents a 55-year-old man with diagnosed GBM who was referred to a CT due to reduced general condition and mild back pain which revealed extensive systemic metastases. A literature review was conducted to identify potential patient or tumor characteristics that may serve as risk factors for metastasis.

**Results:**

ENM from GBM are likely underreported, with limited examples in the literature and low survival rates of only a few months. Certain clinical and histopathological factors, such as male sex, younger age, temporal lobe location, and specific biological markers, have been associated with a higher likelihood of metastasis formation. Bone and/or bone marrow metastases are the most common sites. Despite various treatment regimens being attempted, there is no consensus on the optimal therapeutic approach for this patient group.

**Conclusion:**

Clinical and histopathological factors can aid clinicians in recognizing the potential for ENM in GBM patients. Our review identifies some of the possible patient- and tumor-related risk factors. However, further research is crucial to identify specific molecular markers and elucidate the underlying biological mechanisms that is essential for development of targeted therapies.

## Highlights

• Extraneural metastases (ENM) from glioblastoma are exceedingly rare and there is currently no consensus on treatment strategy.
• Young age, long-term survival, male gender and temporal primary tumor location serve as potential risk factors.
• Metastasis to bone and/or bone marrow is the most common, but several other locations have been reported.
• Alternations in TP53, RB1, PTEN and CDKN2A/B are molecular features associated with development of ENM.
• Clinicians should be aware of the possibility of ENM and initiate proper examinations if suspicious symptoms occur.

## 1 Introduction

Glioblastoma (GBM) represents a highly malignant primary brain neoplasm with a median overall survival of approximately 15 months (Stupp et al., 2005, 2015). Despite extensive research efforts, much remains to be discovered regarding the tumor biology of this entity. The diagnosis is now based on both pathohistological and molecular characteristics. Advancements in treatment modalities are likely to lead to increased patient survival rates. Currently, extraneural metastases (ENM) from GBM are seldom identified and thus have limited clinical significance. The incidence of ENM is estimated to be less than 2% of cases (Kalokhe et al., 2012); however, current epidemiological data is scarce and often outdated. Additionally, the number of cases is likely underestimated, as clinicians seldom actively search for metastasis from GBM, likely due to a lack of awareness about its possibility and the asymptomatic nature of many cases.

We present a rare case of a GBM patient with metastatic spread to multiple organs, including bone, lung, peritoneum, and soft tissue. Additionally, we provide an extensive literature review of similar cases and discuss clinical and molecular findings to explore potential risk factors and mechanisms for ENM.

## 2 Case presentation

**Fig. 1:**
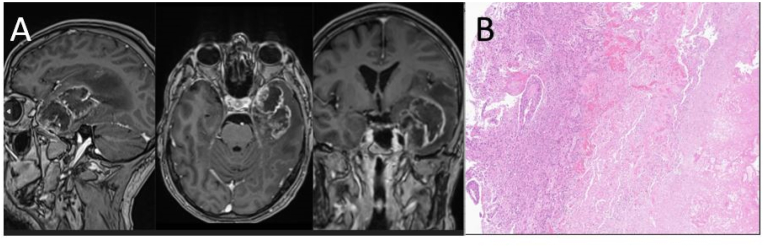
Primary tumor and histology. Patient at initial diagnosis: (A) MRI of the head shows a temporoparietal irregular, inhomogeneous contrast-enhancing tumor with central necrosis and surrounding edema in the sagittal (to the left), transversal (in the middle) and coronal (to the right) planes. The largest diameter measured was 6,5 cm. (B) Histopathological analysis shows tumor cells diffusely infiltrating with areas of necrosis and highly cellular tumor tissue. Hematoxylin-eosin staining; x40.

A 55-year-old male presented with approximately two months history of progressive headache, behavioral change, and concentration difficulties. MRI revealed a left temporal lesion suspicious of GBM (Fig. 1). An initial CT scan of the thorax and abdomen showed no evidence of ENM. Near-total surgical resection was performed using aminolevulinic acid, revealing a hard and dense tumor with necrosis and thrombosed vasculature. The patient made a satisfactory post-operative recovery, experiencing only mild aphasia that progressively improved over the subsequent days. A post-operative MRI revealed a possible minor residual tumor adjacent to the dura at the medial temporal region. Neuropathology confirmed GBM (MGMT promotor methylated, IDH-1/-2- and TERT promotor wild-type) (Table 1).

**Table 1:** Summary of immunohistochemistry and molecular features of our patient.

Primary tumor	IDH-1/-2	Wild-type
	MGMT	Methylated
	TERT promoter	Wild-type
	TP53	Wild-type
	EGFR	Amplification
Paravertebral metastasis	BRAF	Wild-type
	GFAP	Positive
	OLIG2	Positive
	S100	Positive
	CD56	Positive
	NTRK 1-3	No translocation
	PD-L1	Highly upregulated

One month after operation, the patient commenced the Stupp regimen (Stupp et al., 2005) receiving 75 mg/m^2^ temozolomide concurrently with 2 Gy x 30 radiation. Subsequently, he was referred to the local hospital to continue temozolomide adjuvant therapy and completed 6 cycles. The patient tolerated the treatment well.

**Fig. 2:**
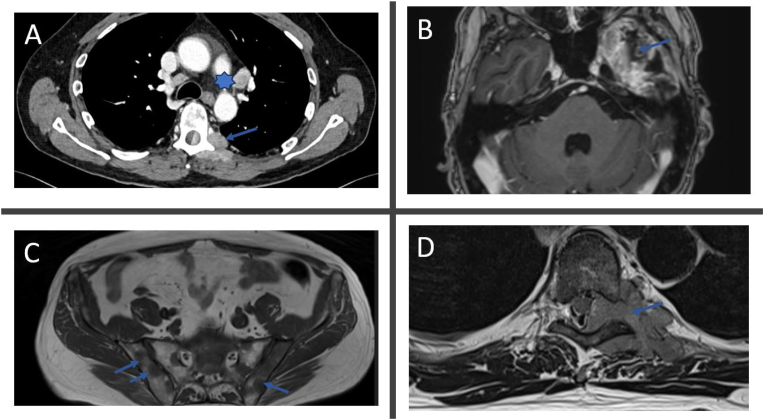
Recurrence. MRI and CT from intra and extracranial progression (A) Transversal CT with contrast shows a large contrast-enhancing paravertebral soft tissue lesion at the left side in the level with Th6. The tumor is growing through the neuroforamina, measuring 5 x 3 cm. Star shows pathological lymph nodes. Arrow shows paravertebral metastasis. (B) Transversal T1 with contrast shows progression of residual tumor tissue basomedial in the temporal fossa. (C) Transversal T1 weighted MR shows multiple hypointense metastatic lesions in the skeleton (sacrum and ilium). Arrows show metastasis. (D) Transversal T2 weighted MRI of the same soft tissue lesion as A. Arrow shows paravertebral metastasis.

**Fig. 3:**
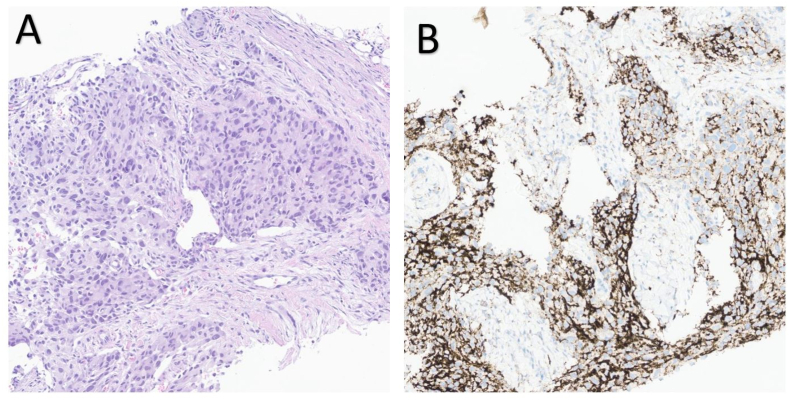
Histology of metastatic lesion. Histology of paravertebral biopsy. (A) Biopsy of the paravertebral lesion showed infiltration of a low differentiated, highly cellular tumor. Immunohistochemically, tumor cells were positive for GFAP, OLIG2, and S100; 100x. (B) Immunohistochemical PD-L1 staining revealed a high percentage of positive cells (>90%) in this specimen; 100x.

Eight months post-surgery, the patient exhibited partial abducens paresis on the left side. Subsequent follow-up MRI scans revealed signs of local tumor progression and involvement of the cavernous sinus, which could potentially explain the palsy (Fig. 2a). The multidisciplinary team at the University Hospital review the patient's case, resulting in a decision to proceed with the continuation of temozolomide and dexamethasone treatment (see Fig. 3).

One month later, the patient was admitted to the local hospital due to a decline in general condition. Infection was suspected, but a subsequent CT scan of the chest and abdomen revealed multiple suspicious lesions in the mediastinum, bones, lung, soft tissue, and mesentery. Additionally, a large lesion was found paravertebral at the level of Th_6_ with growth into the spinal canal (Fig. 2).

The patient was transferred back to the University Hospital, where an MRI of the head showed tumor progression in the left middle fossa, now measuring 69 x 42 x 40 mm. MRI of the thoracolumbar column showed lesions in several vertebrae and a large lesion in Th6, which was biopsied. The biopsy results confirmed that the tumor exhibited histological and molecular characteristics consistent with a metastasis originating from GBM (Table 1). The metastases were treated with radiation with 4 Gy x 5, and the patient showed only mild symptoms (only some back pain) of his ENM upon transfer back to the local hospital.

After commencing a therapeutic combination involving lomustine, vincristine, and bevacizumab at the local hospital, the patient's condition deteriorated rapidly. Within a mere two months, he suffered from his advanced stage of cancer, including thrombocytopenia, hypotension, and tachycardia, ultimately succumbing to systemic failure.

Details on the course, treatment, and outcome are shown in Table 2.

**Table 2:** Summary of our patient.

Patient Sex/age	Oncological history	Molecular biology	Treatment	Outcome
M/55	May 2022: Glioblastoma Grade 4. Jan. 2023: Development of abducens palsy and detection of intracranial progression. Feb. 2023 ENM metastases to bone, soft tissue, lymph nodes, lung, and mesentery.	Primary tumor: IDH-1/-2 wild-type, MGMT methylated, TERT promotor wild-type, TP53 wild-type, EGFR amplification Paravertebral metastasis: BRAF wild-type, NTRK 1-3 no translocation, PD-L1 partly highly upregulated.	May 2022: Neurosurgical debulking of primary tumor. June 2022: 75 mg/m^2^ temozolomide concurrent to 2 Gy x 30 radiation. Aug./Sept.: Temozolomide as adjuvant therapy, six cycles. Feb. 2023: Radiation therapy 4 Gy x 5 against column. March 2023: Lomustine, bevacizumab and vincristine.	Time from diagnosis to death: 11 months. Time from diagnosis to ENM: Nine months Time from ENM to death: Two months.

## 3 Discussion

A comprehensive examination of previous literature regarding metastasis from GBM is summarized in Table 3. While the median or mean age of patients in various studies ranged from 38 to 42 years, our patient was notably older at 55. The studies indicate a mean or median duration from initial diagnosis to metastasis formation spanning from 10 to 26 months, with a median or mean interval from metastasis diagnosis to death ranging from 1 to 10 months. The overall survival duration for GBM patients with metastasis was observed to vary between 7.5 and 26 months. The updated WHO classification, introduced in 2021, redefined GBM criteria, indicating that patients diagnosed before this change could include those with IDH-mutated tumors, associated with a better prognosis (Kumagai et al., 2023). Consequently, studies conducted before 2021 likely included patients with more favorable outcomes.

**Table 3:** Review of the literature.

	Nr	Median or mean age	Localization metastasis	Median or mean survival from diagnosis	Median or mean survival from metastasis	Median or mean time interval from initial diagnosis to metastasis
Piccirilli et al., 2008	128	40	All	17 months	NR	NR
Lun et al., 2011	88	38	All	10.5 months	1.5 months	8.5 months
Kalokhe et al., 2012	79	42	All	13 months	5 months	9.5 months
Pietschmann et al., 2015	150	42	All	13 months	6.0 months	9.0 months
Anghileri et al., 2016	94	NR	All	12 months	NR	8.5 months
Goodwin et al., 2016	28	38.4	Vertebra	26 months	10 months	26.4 months
Cunha and Maldaun, 2019	115	38.2 39.5	All	10/15 months	2.3/4.6 months	7.5/11.7 months
Noch et al., 2021	NR	40	All	11 months	2.5 months	8 months
Strong et al., 2022	92	44	Bone	16 months	3.1 months	NR

NR; not reported.

In our case, metastases were identified nine months following the initial diagnosis, with the patient surviving only two months post-detection of ENM, resulting in an overall survival period of 11 months since the initial diagnosis. These results are consistent with those Noch et al. (Rajagopalan et al., 2005) reported. Interestingly, the overall survival rates for patients with glioblastoma, irrespective of ENM status, remained consistent. (Kalokhe et al., 2012; Rajagopalan et al., 2005). However, a higher number of organ systems affected at the time of ENM diagnosis correlated with a poorer prognosis (Kalokhe et al., 2012). Symptoms of extracranial metastases in glioblastoma can often reflect the specific site of metastasis but may also be vague and non-specific. One of the most detectable signs is the presence of a soft tissue tumor or lymph node metastasis that forms a palpable mass, as observed in cases of metastases to the scalp and neck region, among others (Colwell et al., 2017). Metastases to bone or bone marrow are the most common, and symptomatic patients may experience bone pain or cytopenia due to bone marrow suppression (Axelsen et al., 2007). On the other hand, lung metastasis, another common site, may present with respiratory distress (Voldborg et al., 1997), or in rare cases, it can manifest as acute parotitis (Müller et al., 2014). It's important to note that many extracranial metastases can occur asymptomatically without exhibiting any noticeable symptoms. Therefore, early detection and meticulous monitoring with appropriate screening techniques (Munjapara et al., 2022) are crucial for effectively managing and treating extracranial metastases in high-risk glioblastoma patients.

The review further indicates that the development of ENM is associated with several risk factors, including young age, long-term survival, and male gender. Moreover, ENM exhibits a higher prevalence in primary tumors situated within the temporal lobe, with the parietal lobe following as the secondary most common site (Rajagopalan et al., 2005). Another recent review highlights that the most frequently reported secondary sites for ENM are the bone, lymph nodes, and lungs, as in our patient. Patients with metastases to the lungs generally experience the poorest prognosis (Kumagai et al., 2023). Furthermore, gliosarcoma has demonstrated a greater propensity to form ENM compared to other types of GBM (Noch et al., 2021). GBM with a primitive neuronal component is a rare subtype categorized by WHO in 2016, and fewer than 100 cases have been reported (Kumagai et al., 2023). GBM-PNC with extracranial metastases was first described in 2019 as a metastasis to the lung (Tamai et al., 2019). It has also been detected in the spine (Vollmer et al., 2019) and throughout the skeleton system (Leske et al., 2023). Patients with mutations in MMR could potentially be at a higher risk of developing metastasis. In The Cancer Genome Atlas (TCGA) 499 cases were IDH wild-type and 3% of these had a MMR gene mutation (Kawaguchi et al., 2021). Only 3 cases have shown GBM with MMR gene mutation who metastasize (Kawaguchi et al., 2021; Didelot et al., 2006; Rajagopalan et al., 2005). Thus, these cases are so seldom that there is too early to conclude the potential risk. Understanding these risk factors and patterns of metastatic spread suggests the importance of vigilant monitoring and tailored treatment strategies for patients at higher risk of developing ENM.

The mechanism of metastasis in GBM is not fully understood. Traditionally, surgical intervention and peritoneal seeding via a ventriculoperitoneal shunt have been postulated as rational routes for tumor cell dissemination (Kumagai et al., 2023; Tamai et al., 2019; Vollmer et al., 2019). However, newer reports show metastasizing GBM in cases where neither of these procedures have been performed (Leske et al., 2023), prompting a need to explore the underlying biological aspects. Detection of circulating tumor cells (CTCs) in both blood and cerebrospinal fluid (Kawaguchi et al., 2021; Didelot et al., 2006) indicates that this pathway may also serve as a potential route for metastasis. Further research is warranted to unravel the complex mechanisms driving metastatic spread in glioblastoma and identify potential preventive and therapeutic targets.

Tumor suppressor genes such as TP53, RB1, PTEN, and CDKN2A/B have previously been associated with metastatic glioblastoma (Noch et al., 2021; Chai and Shi, 2022). Additionally, MGMT promotor methylation is more common in the primary site of glioblastoma than in ENM (Zhang et al., 2021; Luan et al., 2021). Studies by Mohme et al. have described that ENM of GBM may involve different genetic drivers and immune escape mechanisms (Mohme et al., 2020). Previous research has identified genetic alterations between the primary lesion and metastasis, indicating an evolutionary process involving subclones (Park et al., 2000).

While evolutional branching in metastases to the brain is well-established (Brastianos et al., 2015), a comprehensive genomic study on metastases from the brain is still needed. Further research is warranted to better understand the molecular features driving the formation of pre-metastatic niches and CTC seeding in foreign tissues. Glioblastoma cells expressing hematopoietic stem cell proteins essential for growth in bone marrow may contribute to bone metastases, which are the most frequent site of metastasis (Noch et al., 2021). Hypoxia-inducible factor-1a and vascular endothelial growth factor (VEGF) play significant roles in this process (Colwell et al., 2017).

Interestingly, GBM has shown overexpression of genes shared with lung and liver cancers (Axelsen et al., 2007), and some CTCs have EGFR amplification (Voldborg et al., 1997; Müller et al., 2014). Zhang et al. found that IDH-mutated grade 4 astrocytoma was as least as likely as GBM to form ENM (Zhang et al., 2021). This may be influenced by the fact that these patients are often younger at diagnosis and tend to have longer survival times. Studies contributing to the growing understanding of the genetic and molecular factors influencing ENM of GBM are essential and may have significant implications for developing targeted therapies and improved management strategies for patients with metastatic glioblastoma.

The screening for BRAF and NTRK alterations yielded negative results (Table 1), ruling out the option of utilizing potential targeted therapies in our patient. A screening for IMPRESS, a national clinical study for targeted therapy, was planned, but insufficient tumor tissue hindered the analysis. The biopsy exhibited a partially high expression of PD-L1, suggesting a potential responsiveness to immune checkpoint inhibitors (ICI). In other cancer forms, ENM has been demonstrated to be linked with a high mutation burden, resulting in higher response rates when treated with immune checkpoint inhibitors (ICI). However, there is also a documented case of a patient who exhibited intracranial stable disease while undergoing ICI treatment but experienced extracranial progression (Colwell et al., 2017). A recent case report about a patient with a BRAF V600E-mutation treated with a combination of BRAF-MEK inhibition showed a response both intracranial and systemic, and progression-free survival for 9 months (Munjapara et al., 2022).

There is no consensus of treatment strategy for patients with metastasizing GBM. Chemotherapy is the only treatment that has shown to prolong the overall survival in metastatic GBM, and various different chemotherapy regimens have been attempted in the past (Kalokhe et al., 2012; Astner et al., 2006). In this specific case, we administered a combination of lomustine, vincristine, and bevacizumab, referred to as LAVA, which is utilized as a last-line treatment for GBM in Western Norway.

A limitation in this case is that only one biopsy was histologically confirmed as true metastasis from GBM. However, it is reasonable to consider that all these metastases were linked to the primary lesion, given the chronological alignment of events and the absence of metastases detected at the time of the primary diagnosis. The tumor was MGMT methylated, which usually means a better prognosis with temozolomide treatment (Leske et al., 2023), but ENM is related to poor prognosis. If we could have recognized the ENM earlier the prognosis might have been better.

## 4 Conclusion

The traditional belief that GBM does not metastasize needs reevaluation. We report a rare case of GBM with extensive extraneural metastases. As overall survival improves, clinicians should consider metastases if relevant symptoms develop. Our literature review suggests that young age, long-term survival, male gender, and primary tumor location are potential risk factors for ENM, though no substantial molecular markers have been identified yet. Further studies on molecular biology and other factors are necessary to pinpoint specific risk factors and mechanisms of ENM. With the new WHO classification, updated studies are needed to characterize ENM epidemiology in GBM patients, guiding future follow-up, treatment, and improving outcomes.

## Ethical statement

The patient gave written informed consent for participating in the study and publishing of the present case report.

## Declaration of competing interest

The authors report there are no competing interests to declare.

## Disclosure-conflict of interest statement

The authors whose names are listed below certify that they have no affiliations with or involvement in any organization or entity with any financial interest (such as honoraria; educational grants; participation in speakers’ bureaus; membership, employment, consultancies, stock ownership, or other equity interest; and expert testimony or patent-licensing arrangements), or non-financial interest (such as personal or professional relationships, affiliations, knowledge or beliefs) in the subject matter or materials discussed in this manuscript.

## Funding details

No funding was sought or awarded.
